# The global significance of Scleractinian corals without photoendosymbiosis

**DOI:** 10.1038/s41598-024-60794-0

**Published:** 2024-05-03

**Authors:** S. Vuleta, S. Nakagawa, T. D. Ainsworth

**Affiliations:** 1 Centre for Marine Science and Innovation, School of Biological, Earth and Environmental Sciences (BEES), The University of New South Wales, Sydney, NSW 2033 Australia; 2 Evolution and Ecology Research Centre, School of Biological, Earth and Environmental Sciences (BEES), The University of New South Wales, Sydney, NSW 2033 Australia

**Keywords:** Marine biology, Biodiversity

## Abstract

Globally tropical Scleractinian corals have been a focal point for discussions on the impact of a changing climate on marine ecosystems and biodiversity. Research into tropical Scleractinian corals, particularly the role and breakdown of photoendosymbiosis in response to warming, has been prolific in recent decades. However, research into their subtropical, temperate, cold- and deep-water counterparts, whose number is dominated by corals without photoendosymbiosis, has not been as prolific. Approximately 50% of Scleractinian corals (> 700 species) do not maintain photoendosymbiosis and as such, do not rely upon the products of photosynthesis for homeostasis. Some species also have variable partnerships with photendosymbionts depending on life history and ecological niche. Here we undertake a systematic map of literature on Scleractinian corals without, or with variable, photoendosymbiosis. In doing so we identify 482 publications spanning 5 decades. In mapping research effort, we find publications have been sporadic over time, predominately focusing on a limited number of species, with greater research effort directed towards deep-water species. We find only 141 species have been studied, with approximately 30% of the total identified research effort directed toward a single species, *Desmophyllum pertusum*, highlighting significant knowledge gaps into Scleractinian diversity. We find similar limitations to studied locations, with 78 identified from the global data, of which only few represent most research outputs. We also identified inconsistencies with terminology used to describe Scleractinia without photoendosymbiosis, likely contributing to difficulties in accounting for their role and contribution to marine ecosystems. We propose that the terminology requires re-evaluation to allow further systematic assessment of literature, and to ensure it’s consistent with changes implemented for photoendosymbiotic corals. Finally, we find that knowledge gaps identified over 20 years ago are still present for most aphotoendosymbiotic Scleractinian species, and we show data deficiencies remain regarding their function, biodiversity and the impacts of anthropogenic stressors.

## Introduction

The successes of modern Scleractinian corals are widely attributed to the evolution of coral endosymbiosis with photosynthetic dinoflagellates (*ref.* photoendosymbiosis)^1,2^ of the newly described family *Symbiodiniaceae* (previously referred to as zooxanthellae)^3,4^. Coral photoendosymbiosis refers to *Symbiodiniaceae* within the peri-algal space of the coral gastrodermal cells, where the products of photosynthesis meet the majority of energy requirements of the host coral colony^5^. Corals (including those with high *Symbiodiniaceae* densities) also rely to varying extents on heterotrophy (filter feeding) to facilitate growth^6^. Coral photoendosymbiosis supports rapid growth and calcification, facilitating their role as foundational species within nutrient deficient shallow water environments^7,8^. As a result, corals are often thought of synonymously with extensive and structurally complex shallow-water tropical reef systems.

However, corals are found across broad biogeographical ranges and thrive in seemingly unlikely environments^9,10^. In fact, approximately 50% of Scleractinian coral species do not maintain photoendosymbiosis with *Symbiodiniaceae*, referred to broadly as azooxanthellate species^11^. Corals without photoendosymbiosis are entirely heterotrophic deriving nutrients from the surrounding water column including organic matter, phytoplankton, and zooplankton^6,12^. Like their photoendosymbiotic counterparts, these corals retain diverse microbial assemblages which may assist in nutrient cycling^6,13^. Although significantly less common, for some corals photoendosymbiosis can be flexible through the coral life cycle, across the coral colony and in response to environmental factors such as light availability^14–16^. These environmentally influenced species are referred to as facultatively symbiotic (previously referred to as apozooxanthellate)^14^.

Corals lacking photoendosymbiosis (*including those referred to as azooxanthellate, apozooxanthallate, and facultatively symbiotic coral species*) together represent approximately half of Scleractinian coral taxa^11^ but often attract generalisations regarding life history traits and ecological role. For example, these species are often referred to as non-constructional (previously *ahermatypic*), not habitat forming, found only in deep or cold-water habitats, and solitary living^17^. However, while many species within these groups align with these descriptions, some have the capacity to form constructional (previously *hermatypic*) colonial reef systems^14,17–19^ and maintain similar calcification rates to photoendosymbiotic species^20^. The absence of photoendosymbiotic dinoflagellates within these coral species removes light constraints on growth, enabling these species to exploit broad geographical and bathymetric ranges. As such, coral species living without reliance on photoendosymbioses are found across polar, temperate, sub-tropical and tropical regions, exhibiting immense depth variation, from the intertidal zone to abyssal depths greater than 6000 m^17,21^. In deep and/or cold-water habitats, coral reefs can play host to highly diverse biological assemblages similar to those of tropical coral reefs^9,18^ but these ecosystems are largely understudied due to the complexities and cost associated with deep-water research, a perceived lack of environmental significance and little information on potential environmental threats^19^. However, these ecosystems cover vast areas, are environmentally important including facilitating speciation within the deep sea^18^ and have significant socio-economic roles, such as hosting breeding grounds for fished species^22^. Importantly, researchers have also suggested that deep-water coral reefs are the most recent ancestor, and direct source of biodiversity, to modern shallow-water tropical reef systems^21^.

Despite their prevalence within the Scleractinian group, corals without or with variable photoendosymbiosis are significantly under-represented within the literature, despite exhibiting broad, globally significant, biogeographic ranges. Here we aim to investigate the global research effort into Scleractinian species lacking photoendosymbiosis (aphotoendosymbiotic) and species with facultative photoendosymbiosis, and in doing so highlight current research gaps to provide future direction within this field. We employ a systematic approach which identifies literature from 1967 (the oldest publication date identified under the developed search string) to 2021 to assess; (1) how terminology has been applied over time to describe aphotoendosymbiotic and facultatively photoendosymbiotic species, their distributions and role within the environment, (2) research output over time, (3) biogeographic patterns in research effort and (4) if the current research effort is reflective of the known diversity of corals without or with variable photoendosymbiosis. Within the context of our findings, we also aim to establish the current knowledge and research gaps surrounding aphotoendosymbiotic and facultatively photoendosymbiotic coral species, including the potential threats and stressors impacting these populations. To achieve these objectives, we use trends identified in the bibliometric and extracted data to inform an extensive review of research effort. In doing so, we identify locations of high publication output for commonly grouped populations of aphotoendosymbiotic and facultatively photoendosymbiotic corals (in the form of shallow, mesophotic and deep-water residing corals) for case study analysis, allowing us to better represent the diversity and variability of research, and establish current knowledge within this field.

## Systematic literature identification protocol

Here we provide the systematic protocol used to identify global research effort into corals without photoendosymbiosis, we also provide detailed systematic descriptions and coding with supplementary material (see all documents within Supplementary File 1: Search Methods Data). We follow recommendations^23^ for systematic protocol of peer reviewed literature, and the ROSES systematic checklist (Supplementary File 1: ROSES Systematic Map Protocols Checklist). Firstly, we undertook a scoping study in Google Scholar utilising common coral symbiosis terminology (such as azooxanthellate, asymbiotic, aposymbiotic, apozooxanthellate, facultatively symbiotic) from which we identified benchmark papers in azooxanthellate research. We generated a list of key words and search terms using publication titles, abstracts and the *wordcloud* package in R studio. These terms, in addition to those found within benchmark papers and other terminology noted throughout the scoping phase, were used to formulate word strings and the inclusion and exclusion criteria used within the study. The search string is provided below and was utilised for searches within; Web of Science, SCOPUS, and Google Scholar (see Supplementary File 1: Search Methods Data for all search result documents) to identify published, peer-reviewed literature dated from 1967 to July 2021. Searches were also conducted using the same search string in ProQuest Dissertation, EBSCO Host Dissertation, and Open Grey, although no results were produced for the latter.

### Search string

(("azooxanthellate" OR "deep sea" OR "deep water" OR "temperate" OR "cold water" OR "apozooxan*" OR "facultat* symb*" OR "aposymbio*" OR "aphotic" OR "asymbiotic" OR "non-symbiotic" OR "heterotrophic" OR "non-photosyn*" OR "non-zooxanthella*") AND ("scleractin*") NOT ("soft coral*" OR "octocor*" OR "black coral*" OR "gorgon*" OR "spong*" OR "gastropod*" OR "clam*" OR "anemon*" OR "shrimp*" OR "oyster*" OR "crustacean*" OR "limestone*" OR "fish*" OR "entobia" OR "foramini*" OR "non-scleractinian" OR "bamboo" OR "worm" OR "lobster*" OR "nudibranch*" OR "crinoid*" OR "mollusk*" OR "barnacle*")).

In total 1242 publications were identified from the systematic procedure, which were then screened for duplicates and inclusion/exclusion criteria within the review following the procedure described here. Publications titles, keywords and abstracts were manually screened for relevance utilising search terms; azooxanthellate, apozooxanthellate, facultatively symbiotic, asymbiotic, non-symbiotic, non-zooxanthellate, non-photosymbiotic, deep and cold. Manual screening, although not recommended, was necessary due to the extensive variability in terminology used within the literature globally. In addition, due to the variability of terminology in this space, and the development of the systematic search string (including inclusion and exclusion criteria, from which some search limitations arise), some relevant articles may not be encompassed within this review (Supplementary File 1: Limitations and Missed Articles). From manual screening, we then compiled a literature database of 482 identified publications (full database provided in Supplementary File 1: Final Database_482 Publications) (Supplementary Fig. 1) from which we undertook a full-text screening to collate the following information;

- Study species
- Study location
- Symbiosis terminology (azooxanthellate, apozooxanthellate, facultatively symbiotic, asymbiotic, non-symbiotic, non-zooxanthellate, non-photosymbiotic, deep and cold)
- Sample and/or species collection depth or study depth (categorised into 0-29 m; mesophotic defined as 30-199 m; and deep-water defined as > 200 m)
- Study ecosystem type (tropical, shallow temperate, cold-water, deep sea),
- Field of research or research discipline (ecology, biology, oceanography), and
- Study authorship, publication type and journal, and year of publication.

Data were analysed within Excel and R Studio to undertake comparisons of research effort, determine areas of high research output, and guide location selection for regional case study analysis. We also screen the final database for titles of relevance to topics of interest (such as threats). Data and coding are openly available at OSF.

## Systematic map of global research effort: results

### Utilisation of terminology

**Figure 1:**
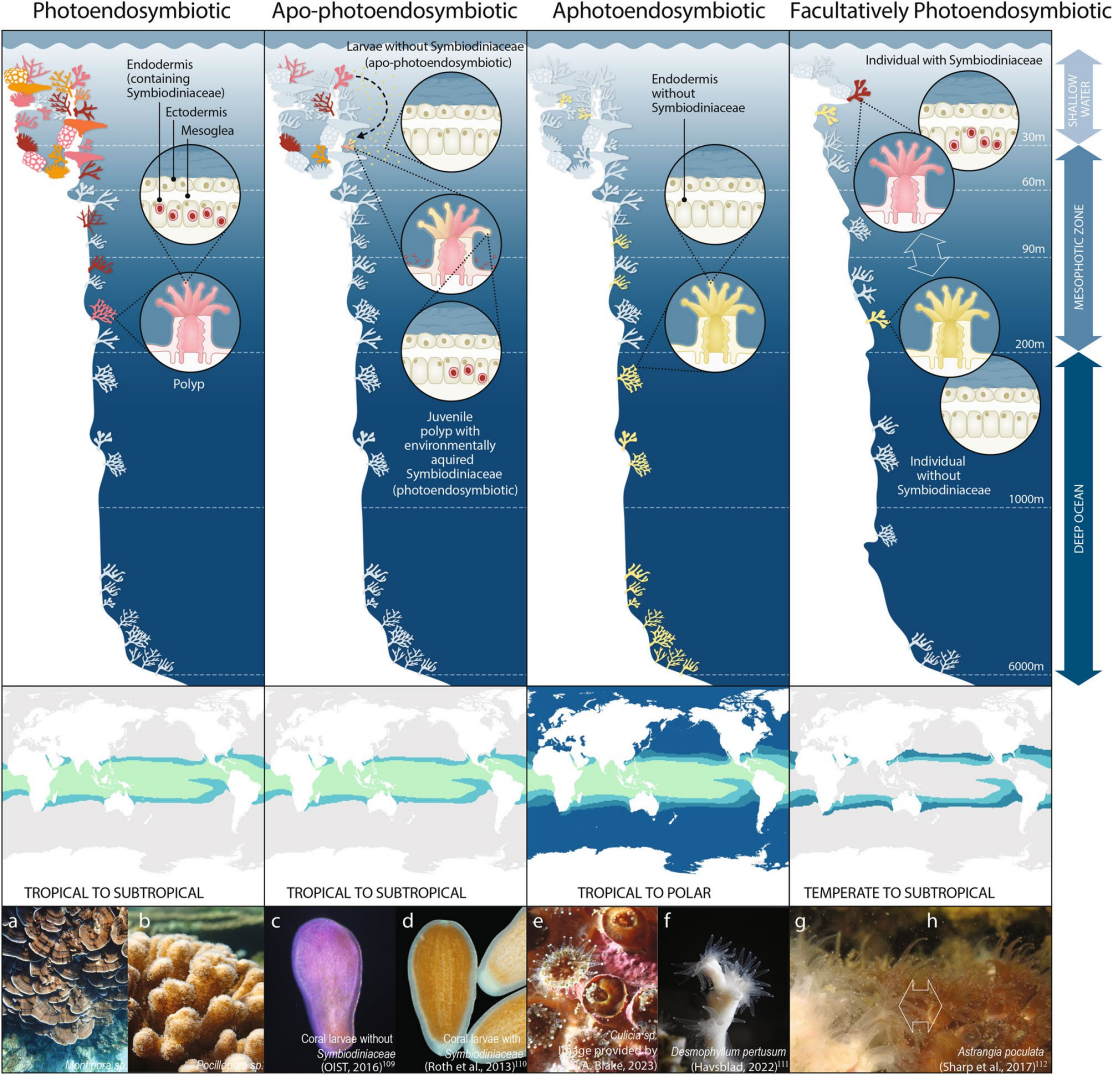
A graphical depiction of the types of coral photoendosymbiosis and their associated biogeographic range extents. Panels (**a**–**h**)^108–111^ are examples of Scleractinian corals from each photoendosymbiosis category. Here we propose updated symbiosis terminology in line with current recommendations within tropical coral science.

**Figure 2:**
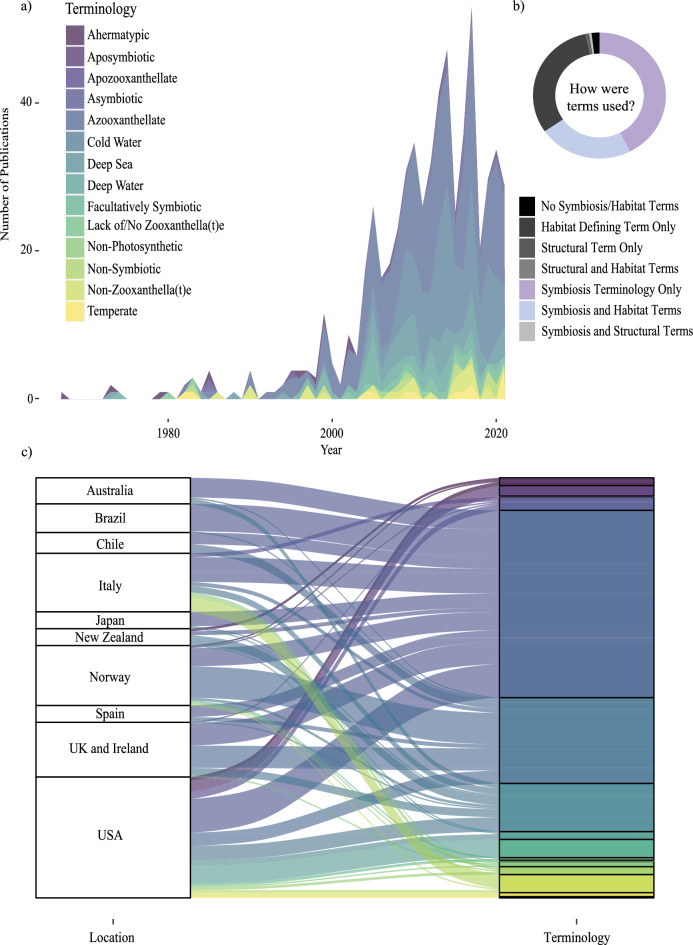
Symbiosis terminology use within 482 publications centred on aphotoendosymbiotic or facultatively photoendosymbiotic coral research was extracted, analysed, and is presented within figure panels a, b and c. Panel (**a**) shows how the identified symbiosis terminology has been used over time, illustrating large variations and inconsistencies within the literature. Panel (**b**) shows how terms were used within the identified publications, and panel (**c**) depicts the relationships between terminology use and regions of high publication output. Overall, our data demonstrates a largely variable and inconsistent use of terminology in the field, and the need for re-evaluation.

Fourteen different terms or phrases were found to describe the absence (or variability) of photoendosymbiotic *Symbiodiniaceae* (zooxanthellae) within a coral host (Table 1, Fig. 1. Figure 2a,b,c). Of these terms, ‘*azooxanthellate*’ was the most frequently applied within the identified publications to refer to the host organism’s photoendosymbiotic state (259 publications (53%)). Habitat defining terms, such as cold or deep sea/water, were also frequently utilised alone to imply endosymbiotic state (*aphotoendosymbiotic*) of the coral species (149 or 31% of publications) (Fig. 2b). Coral species exhibiting environmentally influenced densities of photoendosymbionts, in that the host species has been shown to exist across a spectrum of symbiotic states, were most referred to as ‘*facultatively symbiotic’* (20 publications) but have also been referred to as ‘*azooxanthellate’* and/or ‘*zooxanthellate’* dependant on life stage or environmental conditions, or ‘*apozooxanthellate’*, in 7 publications. The remaining publications used alternative symbiosis terminology, or a combination of symbiotic and habitat defining terminology (Table 1. Glossary of symbiosis terminology; Fig. 2).

**Table 1:** Symbiosis terminology identified within 482 research publications centred on aphotoendosymbiotic and facultatively photoendosymbiotic research from 1967 to 2021, including example and landmark references.

Identified terminology	Definitions and use of terminology	References
*Symbiodiniaceae*	*Symbiodiniaceae *is a family of symbiotic dinoflagellates	^3^
Zooxanthellae	Used in reference to *Symbiodiniaceae* (and the family’s associated genera (previously referred to as clades)) before a systematic revision of the terminology	^4,14^
Azooxanthellate; Non-symbiotic; asymbiotic; Non-photosynthetic; lack of/without/no zooxanthellae	Terms that have been found in reference to coral species that do not form a symbiotic relationship with *Symbiodiniaceae*	^14^
Aposymbiotic/apozooxanthellate	Terms found in reference to one of the following definitions: (1) Coral larva before horizontal acquisition of Symbiodiniaceae (i.e. without *Symbiodiniaceae*); (2) corals that are temporarily free of *Symbiodiniaceae* (i.e. bleaching events); and (3) to describe facultatively symbiotic coral species occurring without *Symbiodiniaceae*	^14,24,27^
Facultatively symbiotic	Reference to coral species found naturally both with and without photoendosymbiosis with *Symbiodiniaceae*. Photoendosymbiosis may be influenced by surrounding environmental conditions	^15^
Cold-water coral	Used to describe corals inhabiting areas outside of the tropics, and commonly used interchangeably or in conjunction with “Deep Water Coral” terminology to define corals occurring at depth	^18^
Deep-water; deep-sea coral	Commonly used to describe corals found at depth (> 200 m) though depth ranges vary	^10,22^
Ahermatypic	Referring to non-constructional or non-reef building corals	^14^
Solitary	Corals that do not form colonies or colonial systems	^14^

Over half of the collated data in the current study utilised the term ‘*azooxanthellate*’ to describe the study species as entirely lacking photoendosymbiosis (*referring to a lack of zooxanthellae; Symbiodiniaceae*). The term ‘*aposymbiotic*’ has been used interchangeably with ‘*azooxanthellate*’ or used to refer to populations of facultatively symbiotic coral communities without *Symbiodiniaceae*^24,25^ or to define facultative symbiosis more broadly^26^. *Aposymbiotic* has also been used to describe the absence of zooxanthellae from the larvae stage of typically zooxanthellate corals^27,28^. Given the variable use of these terms, we aimed to clarify the use of the terminology with the top 50 publication results for ‘*aposymbiotic* coral’ exported from Web of Science and the use of terminology assessed (Supplementary File 1: Aposymbiotic_WOS Top 50). 18% of these publications were excluded on the basis of irrelevance to Scleractinian coral species, 52% used the term ‘*aposymbiotic*’ in reference to a coral larvae life-stage, juvenile coral or the primary adult polyps before the horizontal acquisition of *Symbiodiniaceae*, 18% used ‘*aposymbiotic*’ to describe azooxanthellate populations of facultatively symbiotic species, and 12% of publications referred to the absence of *Symbiodiniaceae* from typically photoendosymbiotic species for experimental purposes, such as bleaching studies. These results together highlight the difficulty in relying upon symbioses terminology for systematic review and meta-analysis of the literature to date and the importance of consistent annotation of symbiotic traits across species.

### Geographic region

**Figure 3:**
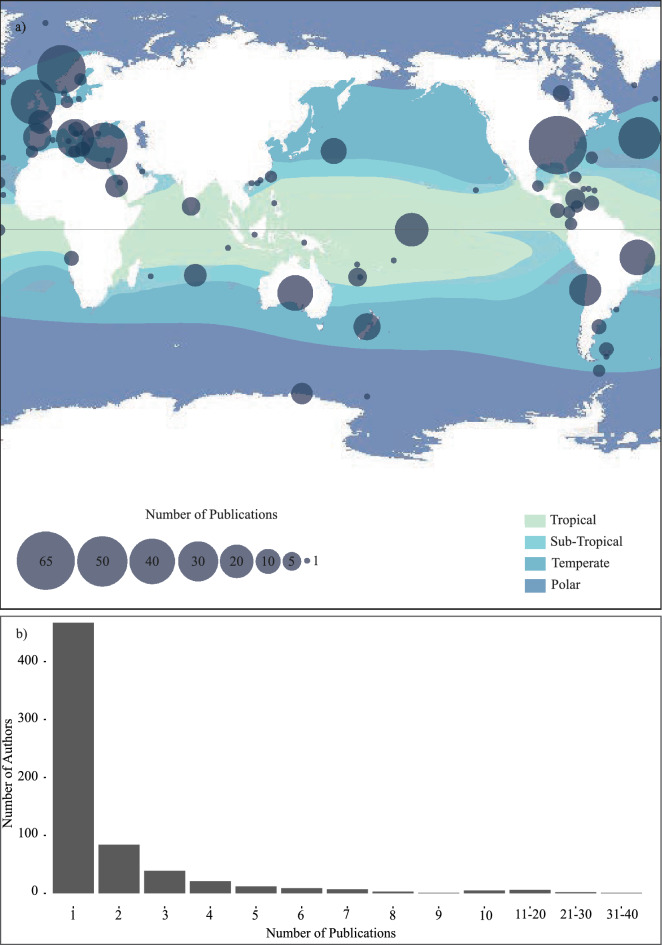
(**a**) A world map illustrating the distribution of identified research effort, with bubble plots indicating the total number of publications identified for each region. Our data shows few locations are responsible for the majority of the research output identified within this review. (**b**) A histogram demonstrating the relationship between identified authors and publication output. Approximately 70% were found to have authored only a single publication in the field.

The study region was recorded as the location of sample collection or (in the absence of samples) the location on which the study was based (Fig. 3a). The highest research effort that was recorded for a geographic region was the United States of America with a total output of 65 publications, followed by Norway with 46 publications, Italy (42) and UK/Ireland (39) (Fig. 3a). We also identify global research effort by ecoregion where applicable (ecoregion definitions by Spalding et al^29^. Study locations are identified as tropical (Lat 23.5 N–23.5 S) (22% of publications), subtropical (Lat 23.5–35 N and S) (5% of publications), temperate (Lat 35–66 N and S) (69% of publications) and polar (Lat 66–90 N and S) (4% of publications), with some studies encompassing multiple climatic zones or ecoregions. Research effort is also noted if conducted in the Global North (70% of identified studied locations) and Global South (30% of identified studied locations) (Fig. 3a). for relevant publications.

### Study authorship

First, second and last authors (determined as the likely leading and senior role authors) were recorded from all 482 publications, with a total of 657 authors identified from the 54 years of identified publications (Fig. 3b). 7% (46) authors were identified to have equal to or greater than 5 publications within the study period and 71% (467) authored only a single publication (Fig. 3b). Cairns SD of the Department of Invertebrate Zoology, Smithsonian Institute, Washington, D.C., Goffredo S of the Marine Science Group, University of Bologna, Italy, Kitahara MV of Centre of Marine Biology, University of Sao Paulo, Brazil, and Roberts JM of Changing Oceans Group, University of Edinburgh, Scotland, were found to be the most prolific researchers leading 39, 27, 22 and 20 of the identified publications respectively.

### Study species (coral)

**Figure 4:**
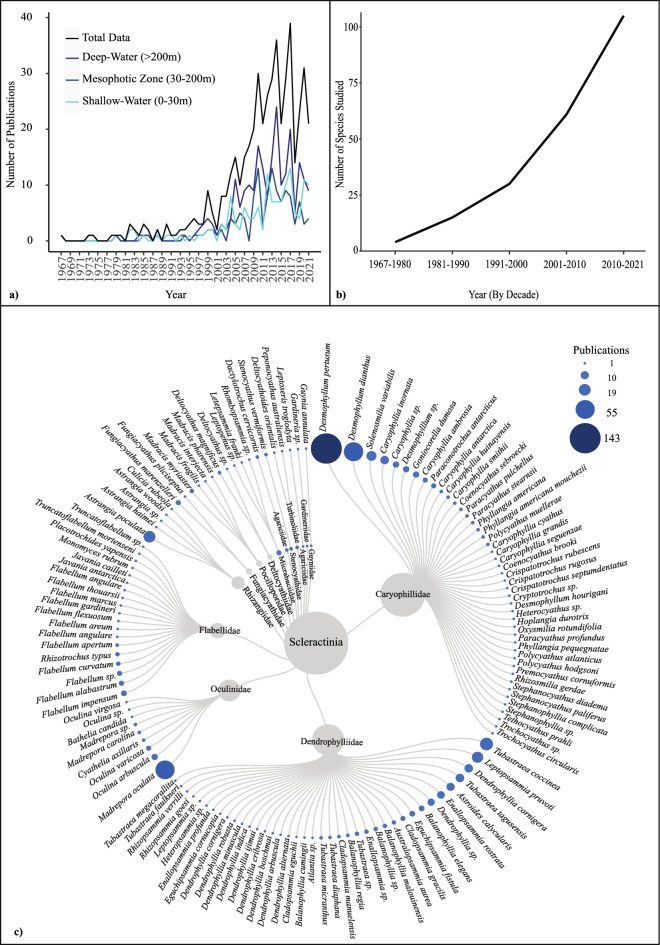
(**a**) Aphotoendosymbiotic and facultatively photoendosymbiotic coral publications from 1967 to 2021 depict a heavily fluctuating research effort over time. (**b**) A representation of the species diversity (number of species) researched over time (by decade) from 1967 to 2021. (**c**) A dendrogram (made using RAWGraphs^112^) of identified study species within our systematic map, with corresponding bubble plots illustrating total research effort (number of publications) for each identified species. Approximately 30% of the identified research effort was attributed to a single deep water, reef-building species *Desmophyllum pertusum*.

Publication dates for the identified literature covered a period of 54 years of research, with the first identified publication in 1967 through to mid-2021. Research effort has increased over time (Fig. 4a,b). Between 1967 and 1998 1 publication was released annually on average, and since 2004 publications have averaged 23 releases annually. However, substantial fluctuations in research effort are evident during the 5 decades of research that has been undertaken. In total 88 of the 482 publications were identified as taxonomic studies, biogeographical studies and/or related to species diversity assessments (Supplementary File 1: Taxonomy and Biogeography Species Diversity Studies). Specifically, taxonomic or record-based publications (59) were found across 36 localities and published at a rate of 0–6 publications per year between 1979 and 2021. Approximately 72% of the 482 identified studies focused on species found in waters greater than 30 m (encompassing mesophotic and deep-water habitats), and 28% of publications focused on shallow water residing species (< 30 m) (Fig. 4a) excluding publications where depth range was not available or applicable.

A total of 141 species of aphotoendosymbiotic or facultatively symbiotic Scleractinia have been studied from 482 publications (excluding record-based publications (see Supplementary File 1: Species Data) (Fig. 4c). Species diversity (i.e. the number of species studied) was found to increase over time (Fig. 4b). However, species were typically limited to 1 publication (58%) and only 14% (20 species) were studied in greater than 5 publications across all ecosystem types. The highest research output has been dedicated to the deep-water coral *Desmophyllum pertusum*^30^ (Linnaeus 1758; Addamo et al. 2016) (previously; *Lophelia pertusa*^30^) which was recorded within 143 publications, accounting for approximately 30% of the dataset generated in the current study (Fig. 4c). A substantial research effort has also been directed to *Madrepora oculata* and *Desmophyllum dianthus*, both accounting for approximately 12% of total publications respectively. *Tubastraea coccinea*, *Astrangia poculata* (previously *A. danae*) and *Leptopsammia pruvoti* were the predominant shallow-water species within the datum, cited within 25, 21 and 19 papers respectively (approximately 5% of the dataset) respectively (Fig. 4c).

### Species depth

The depth data of studied species, including those collected within in situ sampling, was also collated and categorised as either shallow (defined as 0-29 m), mesophotic (defined as 30–199 m) (encompassing potential variation in depth limits within the literature), and deep-water residing (defined as > 200 m) as per previously published depth ranges^31^. Where the identified publications encompassed multiple depth categories, all relevant categories were recorded (See Supplementary File 1: Final Database_482 Publications). We find a consistently greater research effort for species collected from deep-water habitats (47% of publications) compared to those collected from mesophotic (25%) and shallow water (28%) (Fig. 4a), although for all depth categories we find an overall increase in research output over time despite heavily fluctuating output per year (Fig. 4a).

### Case study analyses

**Figure 5:**
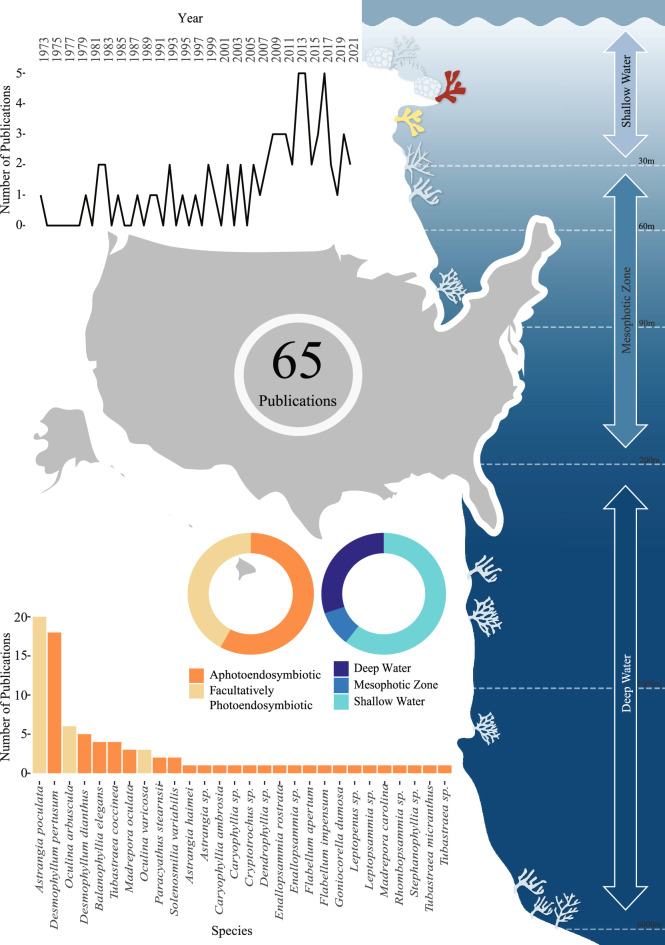
Total identified research output for aphotoendosymbiotic and facultatively photoendosymbiotic corals in the United States of America. The line graph represents the number of publications over time for this location. The donut plots shows both; the research effort for aphotoendosymbiotic and facultatively photoendosymbiotic species (in shades of orange) and the identified research effort for each of the defined bathymetric zones (in shades of blue) (with depth ranges detailed within the right panel). The bar chart illustrates the species studied within this location, and the number of identified publications associated with each species.

**Figure 6:**
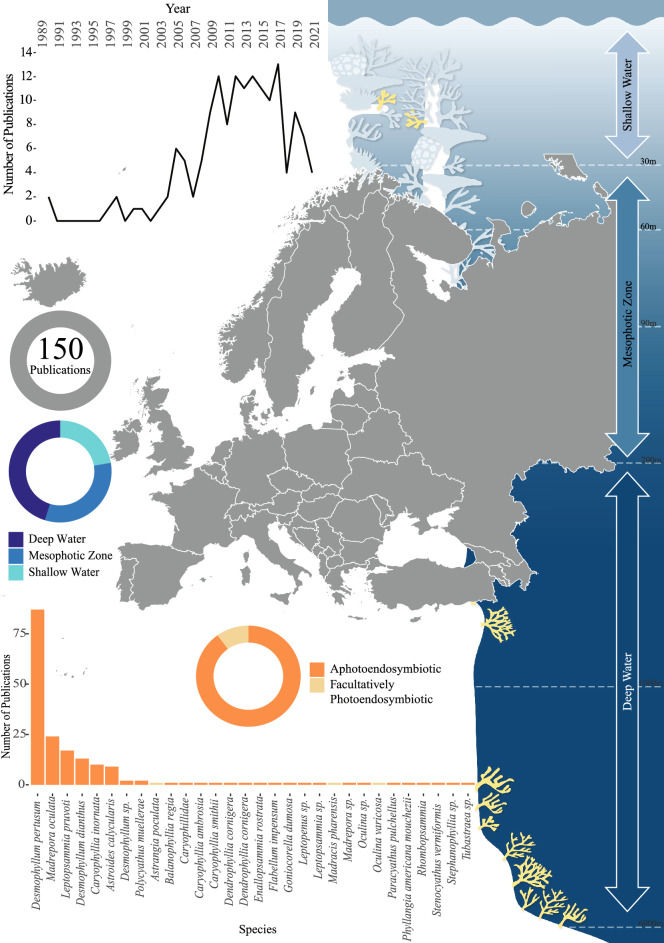
Total identified research output for aphotoendosymbiotic and facultatively photoendosymbiotic corals across Europe. The line graph represents the number of publications over time for this location. The donut plots shows both; the research effort for aphotoendosymbiotic and facultatively photoendosymbiotic species (in shades of orange) and the identified research effort for each of the defined bathymetric zones (in shades of blue) (with depth ranges detailed within the right panel). The bar chart illustrates the species studied within this location, and the number of identified publications associated with each species.

Here we group research effort by the most studied geographic locations (by exclusive economic zone) for shallow, mesophotic and deep-water residing aphotoendosymbiotic and facultatively photoendosymbiotic coral species (using geographic region data). These include the United States of America (USA) (65 publications) (Fig. 5), Europe (150 publications) (Fig. 6) (of which 28% of publications were undertaken in Italy (Supplementary Fig. 2), 31% undertaken in Norway (Supplementary Fig. 3)), and the United Kingdom and Ireland (39 publications) (Supplementary Fig. 4).

#### United States of America

Studies grouped under the ‘USA’ region depicted the highest number of publications globally, totalling 65 papers. Depth data from these publications illustrated a comparatively heightened focus on shallow water facultatively symbiotic Scleractinian corals, (27 publications) (Fig. 5). A total of 28 species were identified from these studies, however *Astrangia poculata* (previously *A. danae*), *Desmophyllum pertusum*, *Oculina arbuscula*, *Desmophyllum dianthus* and *Balanophyllia elegans* formulated approximately 62% of the dataset. *Astrangia poculata* and *Oculina arbscula* alone represent approximately 31% of the identified research effort within the USA, despite facultatively symbiotic species only comprising 6% of global research data within this review (Fig. 5). Most studies grouped under USA focusing on *Astrangia poculata* were located within waters surrounding Rhode Island (Fig. 5).

#### Europe

Ocean and coastal habitats within the regional group of Europe included 150 research publications, with research effort peaking between 2013 and 2017. Research effort was found to be dominated by studies of *Desmophyllum pertusum* (> 50% of the publications) and deep-sea ecosystems (Fig. 6). Within the European region, studies associated with Italy accounted for 42 publications and Norway 46 publications.

##### Italy

Studies conducted along the Italian coastline (grouped under ‘Italy’) focused predominantly on shallow water (< 30 m depth) azooxanthellate corals, with 42 of the identified publications centred on shallow water research (2005–2021) (comparable to the shallow water research output from the USA). *Leptopsammia pruvoti*, *Caryophyllia inornata* and *Astroides calycularis* were the most studied species, at 43%, 26% and 18% of total publications respectively (Supplementary Fig. 2).

##### Norway

In Norwegian waters research predominantly focused on *Desmophyllum pertusum* across both mesophotic and deep-water habitats (Supplementary Fig. 3).

#### UK and Ireland

In total, 39 publications come from studies within UK and Ireland marine and coastal habitats, with research focused on reported ‘azooxanthellate’ species predominantly undertaken in deep sea (56%) and mesophotic (45%) habitats (Supplementary Fig. 4).

#### Global South

**Figure 7:**
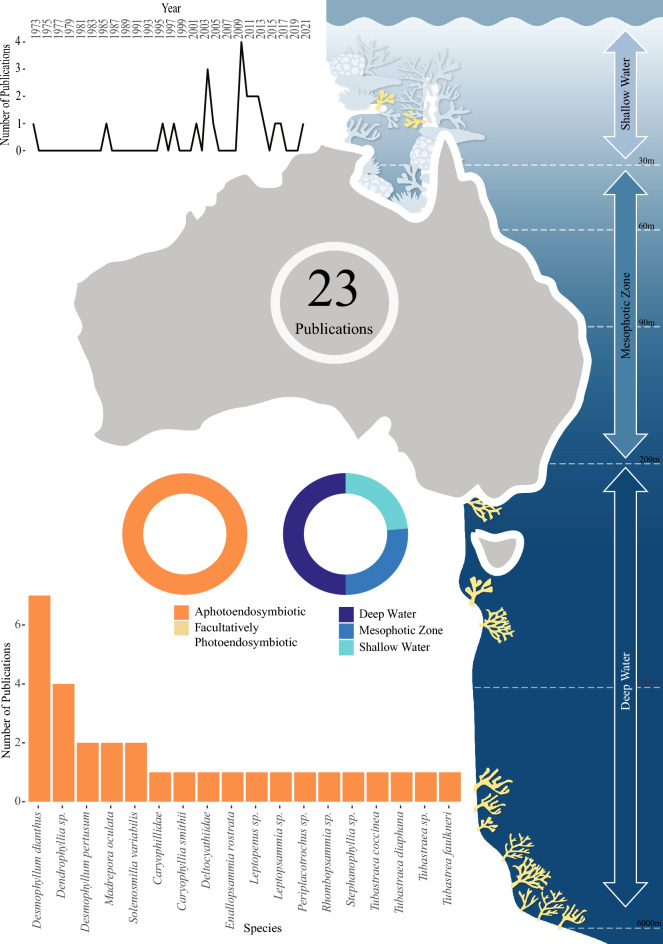
Total identified research output for aphotoendosymbiotic and facultatively photoendosymbiotic corals in Australia. The line graph represents the number of publications over time for this location. The donut plots shows both; the research effort for aphotoendosymbiotic and facultatively photoendosymbiotic species (in shades of orange) and the identified research effort for each of the defined bathymetric zones (in shades of blue) (with depth ranges detailed within the right panel). The bar chart illustrates the species studied within this location, and the number of identified publications associated with each species.

The Global South represents 22 of the 78 studied locations globally (Fig. 3a). In South America, Chile (18 publications) was the most studied location, together with Australia (23 publications) and New Zealand (12 publications), representing the majority of Global South based research effort. In Australia (Fig. 7) 23 publications have focused on few species (18 species) with only 5 study species included in greater than 2 publications over 5 decades of research.

## Discussion

Bibliometric and extracted data from 482 publications centred on aphotoendosymbiotic and facultatively photoendosymbiotic research was analysed with the objectives of detailing inconsistencies associated with the use of symbiosis terminology, research output over time, biogeographic patterns in research effort, and if the current research effort is reflective of the known diversity of corals without or with variable photoendosymbiosis. Here we discuss our findings in relation to these objectives by detailing; the importance of consistent terminology; the global research effort to date for aphotoendosymbiotic and facultatively photoendosymbiotic Scleractinian corals; species and habitat specific trends in the data; and current knowledge in the field. The latter is discussed by defining habitat and location specific trends in the data, to inform shallow water, mesophotic and deep-water case study analyses. In doing so, we identify large research gaps and provide future research direction within the field.

### Terminology

Importantly our study has highlighted there is substantial variability in the use of terminology surrounding corals without photoendosymbioses (Fig. 2). Different locations and ecosystems studied over the past 5 decades have used a variety of terms to refer to, or infer, the endosymbiotic state of the study organisms. We also note that the terminology has not yet changed in line with the taxonomic revision of the photoendosymbiotic dinoflagellate group^3^, which was previously referred to as zooxanthellae, a term now replaced in tropical coral literature with the dinoflagellate family name *Symbiodiniaceae*.

The application of a systematic methodology undertaken here highlighted the need for a re-evaluation of the terminology surrounding corals that lack photoendosymbioses. Scientific terminology evolves alongside increasing knowledge and expertise, and we find use of terms in what was ‘*azooxanthellate*’ coral literature has varied both over time and across the study locations. We further identify a need to update terminology to reflect recent changes in both coral and symbiosis research. As early as 1985^14^ a redefinition of the ecological groups of corals was published, aiming to address inaccuracies of utilising terms related to (or inferring) coral symbiosis^14^. Specifically, the seminal review of the ecological groups of corals^14^ addressed the use of hermatypic (constructional; reef-building) and ahermatypic (non-constructional; non-reef building) to infer presence or absence of zooxanthellate endosymbiosis within Scleractinia coral species. As a result of the work terminology shifted from ‘ahermatypic’ which was the predominant term used in publications from 1967 to 2000, to ‘*azooxanthellate*’ having used extensively from 2000 until present day, to refer to Scleractinia lacking photoendosysmbiosis. However, despite Schuhmacher and Zibrowius’ (1985) review^14^, confusion about the terminology surrounding ‘azooxanthellate’ and facultatively symbiotic coral species continued to be evident in the literature. This confusion is likely due to the variability within and between species in the uptake and maintenance of photoendosymbiosis within the host organism’s life cycle, across species, and across evolutionary timescales for some species. For example, of the fourteen different terms or phrases identified with literature of the past 5 decades to describe Scleractianian (coral) species that are functionally normal in absence of photoendosymbiosis with dinoflagellates (*syn*. zooxanthellae; family *Symbiodiniaceae*) (Table 1) four habitat-defining terms (such as cold-water or deep-water coral) were used to infer the absence of a photoendosymbiosis within a species, habitat, or life stage (Fig. 1). Several publications were also found to have utilised multiple symbiotic terms within single publications (i.e. azooxanthellate, non-photosynthetic and asymbiotic) to refer to the symbiotic nature of the study organism. For example, we find the term ‘*aposymbiotic*’ has been predominantly used to describe the absence of zooxanthellate from coral larvae or juvenile life stages in coral species that environmentally (horizontally) acquire *Symbiodiniaceae* post-settlement, but it has also applied more generally to species without symbiosis, species that have facultative symbiosis, or species that are temporarily free of photoendosymbionts (such as bleaching experiments). Inconsistent use of terminology limits the accessibility of the research^32^, making evidence syntheses challenging.

Importantly, within the coral literature of the past decade, and in response to the re-evaluation of taxonomy of dinoflagellates, the terms ‘*zooxanthalla(e)*’ or ‘*zooxanthellate*’ are no longer used to describe the dinoflagellate in photoendosymbiosis with corals and have been replaced by the family name *Symbiodiniaceae*^3^. Within the family *Symbiodiniaceae*, what was previously referred to as zooxanthellae clades are now classified as genera (*Symbiodinium, Breviolum, Cladocopium, Durusdinium, Effrenium, Fugacium, Gerakladium)*^3^. As such the term azooxanthellate (denoting a lack of zooxanthellae) is also no longer relevant when referring to the absence of photoendosymbiosis. Furthermore, literature investigating other microbial symbioses of corals have also expanded significantly within the past decade with the term ‘*symbiosis*’ now widely used to encompass not only intracellular photoendosymbiosis^33^, but also bacterial symbiosis^34^ and symbiotic microbial eukaryotes within the coral skeleton (endoliths)^33^. Interestingly some studies have also found coral species unexpectedly maintain intracellular dinoflagellates in low (or no) light habitats that do not support photosynthesis^35^. In these environments the endosymbionts have an unknown functional role and are hypothesised as parasitic and not symbiotic^35^. Therefore, differentiating functional photoendosymbiosis (with dinoflagellates of the family *Symbiodiniaceae*) from other symbiosis (such as epi- or ecto-symbiosis; meaning to live on the surface of an organism, as opposed to the intracellular relationships discussed here) is also important in the coming years as research and the tools available to investigate symbiosis become more widely applied. Therefore, here we suggest the use of *photoendosymbiosis* over *symbiosis* in referring to dinoflagellate symbiosis due to the increasing research effort into the breadth of symbioses of corals and the likeliness for future confusion of terminology referring to prokaryotic and eukaryotic symbioses, and epi and endo symbioses of corals. We therefore suggest revising terminology that describes the many different symbiotic states of corals and clearly refer to the symbioses in relation to the interaction with the family *Symbiodiniaceae* (Table 2).

**Table 2:** Our recommendations for standardising coral symbiosis terminology based on (1) updates to symbiosis terminology within photoendosymbiotic (predominantly tropical) coral science and (2) inconsistent terminology use identified throughout 482 publications centred on aphotoendosymbiotic and facultatively photoendosymbiotic coral research.

Revised terminology	Definition
Photoendosymbiotic	Corals with endosymbiotic photosynthetic dinoflagellate algae (*Symbiodiniaceae*)
Aphotoendosymbiotic	An updated term in line with revisions by^3^ to describe corals without photosynthetic dinoflagellate algae (*Symbiodiniaceae*)
Apo-photoendosymbiotic	The absence of *Symbiodiniaceae* from the larval stage of typically photoendosymbiotic coral taxa (i.e., before horizontal acquisition)
Facultatively Photoendosymbiotic	Coral taxa demonstrating natural variance in photoendosymbiosis with *Symbiodiniaceae* (i.e. coral taxa that are able to exist on a spectrum of photoendosymbiosis; from photoendosymbiotic to aphotoendosymbiotic). This variance is commonly influenced by surrounding environmental conditions
Habitat defining terms (i.e. cold-water, deep-water, temperate)	Habitat defining terms should be used in conjunction with symbiosis terminology, not to infer symbiotic state alone, due to the exceptions and inconsistencies highlighted throughout this review

### Terminology recommendations

For corals exhibiting intracellular symbiosis (*endosymbiosis*) with photosynthetic (*photo*) algae of the family *Symbiodiniaceae*, we recommend the term *photoendosymbiotic,* in line with revisions by LaJeunesse et al^3^.

#### Aphotoendosymbiotic, or asymbiotic with Symbiodiniaceae

We suggest ‘***aphotoendosymbiobiotc’*** (lacking photosynthesising endo- (intracellular) symbionts (*Symbiodiniaceae*)) or asymbiotic with *Symbiodiniaceae*, to be used to describe the absence of *Symbiodiniaceae* photoendosymbiosis with a coral species throughout all stages of the host life cycle. In doing so we also recommend that geographical and bathymetric ranges (for example “cold-water coral” or “deep-sea coral”) not be used interchangeably with symbiotic terminology, but in addition to, as the range extents of symbioses within a host species can vary greatly (as shown in Fig. 1) (similar issues were outlined by^14^ in discussions of the term ‘hermatypic’ to group species).

#### Apo-photoendosymbiotic or apo-symbiotic with Symbiodiniaceae

The term ‘*Apo-symbiotic’* was most used to infer the absence of *Symbiodiniaceae* from the larval stages of photoendosymbiotic corals prior to horizontal acquisition of *Symbiodiniaceae*. We therefore suggest that the term ‘***apo-photoendosymbiotic***’ or ‘***aposymbiotic with Symbiodiniaceae***’, more accurately refers to the horizontal uptake of *Symbiodiniaceae* (i.e. photoendosymbionts are acquired from the environment post-settlement). Additionally, these terms may also be applicable to corals that are temporarily free of symbiosis^14^ for other reasons (such as typically photoendosymbiotic corals being experimentally manipulated to be apo-photoendosymbiotic, such as in bleaching studies).

#### Facultatively photoendosymbiotic

The term ‘*apo-zooxanthellate*’^14^ was presented to describe corals that are temporarily free of zooxanthellae for a variety of reasons (also referred to as ‘*aposymbiotic*’). ‘*Apozooxanthellate*’ has since been applied to corals that exhibit a facultatively symbiotic relationship with zooxanthellate^36,37^. However, more recent literature^15^ has highlighted that facultative symbiosis exists on a spectrum across high to low *Symbiodiniaceae* densities which is largely influenced by surrounding environmental conditions (such as light, irradiance and temperature), regardless of developmental stage. Here we found the term ‘*facultatively symbiotic*’ was twice as likely to be utilised in place of ‘*apozooxanthellate*’ in the scientific literature and used interchangeably with different life history traits of the host species. We further suggest use of ‘**facultatively photoendosymbiotic**’ to describe species of coral that occur naturally both with and without photoendosymbiosis as the result of environmental influence.

Facultatively photoendosymbiotic species that have gained, lost, and regained symbiosis through evolutionary timescales can then also be differentiated by these events, as these events are likely to be driven by environmental conditions. In referring to species that lack photoendosymbiosis but are known to come from an evolutionary background of maintaining symbioses, we suggest referring to these species as ***aphotoendosymbiotic*** (or asymbiotic with *Symbiodiniaceae*) and evolutionarily facultative. However, species with variable symbioses due to environmental influence should be referred to as ***facultatively photoendosymbiotic*** across evolutionary history. Categorising species as facultative across evolutionary timescales may aid in denoting which species within the diverse Scleractinia group have undergone environmentally facilitated symbiotic events.

### Timeline of global research effort

Research into corals without photoendosymbiosis (terms azooxanthellate, apozooxanthallate, or the 14 terms or phrases identified), has increased over the near 5 decades of research effort in this field (1967–2021) with effort into deep water, shallow water, tropical and temperate research fluctuating extensively. Interestingly, some of the observed publication peaks may align with conferences, workshops, or other scientific initiatives within the field (Supplementary File 1: Scientific Initiatives). While some similarities in research effort across bathymetry may also be attributed to accessibility of the ecosystems and advancing technology allowing access to remote ecosystems, the overall trend highlights inconsistent research effort, even for shallow water aphotoendosymbiotic corals.

According to Cairns^38^ there are over 711 identified species of extant Scleractinia without photoendosymbioses. However, of the known 711 species, only 21% were recorded within the collated literature of this review. Further to this, 61% of species identified within the review data were studied once (1 publication), demonstrating a superficial knowledge of those identified within the literature. Approximately 30% of the total publications identified here focused on the deep-sea reef foundational coral *Desmophyllum pertusum,* with a further 20% reporting on 2 other deep-sea corals *Madrepora oculata* and *Desmophyllum dianthus*. These three deep-sea residing species alone encompass over half of the research effort identified for aphotoendosymbiotic species. In 2001, Cairns^17^ attributed increases in the taxonomic literature to deep-sea discoveries and advancements. This trend is still apparent today with deep-water research dominating research effort despite approximately one third of corals in these functional groups exploiting shallow-water environments or intertidal zones^17^. *Tubastraea coccinea* and *Astrangia poculata* (previously *A. danae*) were the dominant shallow-water species, here reported within 25 and 22 papers (approximately 5% of the dataset) respectively, illustrating these research gaps extend across all ocean habitats. Further to this, the most studied species are found to occur in close proximity to regions of high publication output (such as *A. poculata* and the USA).

Over 80% of the coral species identified in Cairns’ review of azooxanthellate taxonomic research^17^ were described by just 20 taxonomists and researchers, highlighting disproportional scientific effort to potential of the aphotoendosymbiotic and facultatively endosymbiotic coral groups^17^. The identification of first, second and last author data from 482 relevant papers identified a total of 657 authors of which 467 (approximately 71%) authored only a single publication. A total of 15 authors (cited as either first, second or last) exceeded 10 publications within the dataset, with only 3 researchers surpassing 10 publications as first author. Considering the overall scarcity of studied locations coupled with the immediate impact of marine ecosystem decline on coral reefs, our study also highlights the potential for extensive biodiversity losses in under- and un-studied ecosystems. Finally, 50% of the identified publications in the current study were greater than 10 years old, highlighting the urgent need for continued research effort within this field. 243 studies have been published globally in the last 10 years with a bias in research effort towards specific species (75 of the 243 studies on top 3 studied species) and locations, with only 78 locations studied worldwide and the majority of articles of the last 10 years focusing on deep-water biodiversity assemblages. Taken together this analysis further illustrates large knowledge gaps associated with shallow, temperate, cold, and deep-water systems despite these corals illustrating comparable species level diversity and similar threats to tropical corals.

### Habitat and location specific research effort global trends

Coral species exploit depths that far exceed the photic zone and occupy environmental niches unavailable to their shallow water counterparts as the absence of photoendosymbionts removes light constraints associated with photosynthesis. Aphotoendosymbiotic coral species reside in a variety of habitats including as cryptic species in tropical coral reefs, and can play important ecological roles, including habitat formation. Species may be exclusively shallow water residing, such as *Astroides calycularis*^39^, persist over broad bathymetric ranges (intertidal to deep)^40^, such as *Leptopsammia pruvoti* and *Caryophyllia inornata*^39^, or be confined to greater depths, such as *Desmophyllum pertusum*.

### Case study analyses

Case study analyses were established by identifying biogeographic patterns in the data to inform shallow, mesophotic and deep-water case studies. Here we present habitat and location specific summaries of the current knowledge surrounding aphotoendosymbiotic and facultatively photoendosymbiotic species to address key aims, including research output over time, biogeographic patterns in research effort, and if the current research effort is reflective of the known diversity of aphotoendosymbiotic and facultatively symbiotic coral species. In doing so, we highlight relevant research gaps and future research directions.

#### Research effort in shallow-water habitats (0-30 m)

Interestingly only one study^40^ was identified to review shallow water ‘azooxanthellate’ Scleractinia as a group and the review was confined to the western Atlantic locations. In the review Cairns^40^ highlighted 73 shallow-water ‘azooxanthellate’ species for the study region and alluded to the potential biodiversity globally for shallow water corals lacking photoendosysmbioses. Some coral species are facultatively photoendosymbiotic with *Symbiodiniaceae* in that environmental factors, such as light, irradiance or temperature, influence the relationship between host and photoendosymbiont^15^, resulting in a spectrum of symbiotic interactions^41,42^. The variability in photoendosymbioses as a factor of environment is evident between and within the individual host coral colonies, between shallow and deeper water habitats, as well as between colonies in proximity^43^. Very few publications into facultatively symbiotic corals were identified in the current study with only 6% of the total identified studies specifically addressing corals with variable photoendosymbioses (facultative photoendosymbiosis). 11 facultative species were outlined in one of the earliest reviews of Scleractinian species diversity^11^, while the Coral Trait Database (CTD) was found to be the only other resource to list facultatively symbiotic corals, currently citing 12 species of Scleractinian coral to be facultatively photoendosymbiotic, representing 0.8% of the coral database^44^. Here we find only two facultatively symbiotic species *Astrangia poculata* and *Oculina arbuscula* have been studied in detail, representing 4% and 1% of the total literature identified. The relatively minimal scientific attention directed toward facultative photoendosymbioses raises the question as to whether this form of symbiosis is as uncommon as assumed or if improved research effort would provide a better understanding of rates of facultative photoendosymbioses, or symbiotic variability, worldwide. For example, *Oculina arbuscula* is currently not recognised as a facultatively symbiotic species on the CTD (however is recognised in the published literature^45^). *Astrangia poculata* has a broad biogeographical range with species records in USA spanning over 2000 km, from approximately Cape Cod, Massachusetts, to the Gulf of Mexico^46^. The wide-spread geographical success of these corals, particularly *A. poculata*, suggests a broad environmental resilience^46,47^. For example, Rhode Island populations of *A. poculata* alone withstand seasonal temperature variations ranging from 0 to 27 degrees Celsius^25,48^. Publications by Goffredo et al. (2010) and Caroselli et al^49,50^. were amongst the first to explore these topics for ‘azooxanthellate’ Scleractinia, demonstrating species to exhibit homogeneity of growth patterns despite changes in surface radiation and temperature associated with latitudinal variation, suggesting the local adaptation potential for shallow water corals with variable photoendosymbiosis. The adaptation potential and resilience of these species is of increasing interest, as is the role of facultative photoendosymbioses in coral populations withstanding substantial ecosystem changes associated with climate change. However, the potential for these species and their resilience to climate change across habitats is not well known due to substantial knowledge gaps. To date, facultatively symbiotic species have only been studied within shallow waters (0–30 m) in sub-tropical environments and these species are poorly understood outside of these regions, with *Astrangia poculata* and *Oculina arbuscula* the only identified facultatively photoendosymbiotic species in the current study.

*Astrangia poculata* has been found to occur in association with *Symbiodiniaceae,* species *Breviolum psygmophilum*^51^, which are horizontally acquired by the coral larvae post-settlement^52^. Several comparative studies have been conducted on ‘zooxanthellate’ and ‘azooxanthellate’ populations of *A. poculata*^15,16,41,51,53,54^. Initial studies demonstrated a correlation between increasing temperature and the calcification rates of both ‘zooxanthellate’ and ‘azooxanthellate’ colonies of *A. poculata*^41^ and *O. arbuscula*^42^. Similar trends have been identified for *A. poculata*^15^, correlating temporal variations in ‘zooxanthellate’ or chlorophyll densities to seasonal changes. A later paper suggested that photoendosymbiotic *A. poculata* colonies facilitated increased densities of *Symbiodiniaceae* within the warmer months when symbiosis was of benefit (maximising growth rate), and reduced population densities under less favourable conditions (temperature decline) when the symbiotic relationship was of greater cost to the host^16^. Interestingly, colonies presumed aphotoendosymbiotic illustrated similar (although less distinct) temporal fluctuations in *Symbiodiniaceae* densities^15^. This was attributed to the concept that some aphotoendosymbiotic corals may not be strictly devoid of *Symbiodiniaceae*, enabling minute populations to increase under favourable conditions^15^. The limitations of conventional molecular techniques in detecting low densities of endosymbiotic *Symbiodiniaceae* have been discussed^55^. However, misconceptions surrounding aphotoendosymbiotic and facultatively photoendosymbiotic coral holobiont composition is more likely attributed to a lack of understanding comparative to photoendosymbiotic coral species, facilitating assumption-based aphotoendosymbiotic categorisation based on pigment or depth profile alone. For example, Wagner et al. (2011) established Hawaiian black corals sampled beyond the photic zone (up to ~ 400 m) retain endosymbiotic *Symbiodiniaceae*, further alluding to the diversity of some dinoflagellate species^35^.

In contrast to their varying associations with *Symbiodiniaceae*, the importance of heterotrophy has been discussed for temperate facultatively symbiotic corals *O. arbuscula* and *A. poculata*. For example, the feeding ecology of *O. arbuscula* has previously been compared for both photoendosymbiotic and aphotoendosymbiotic coral communities^24^. Previous work by Leal et al^24^. has shown the importance of the pico- to nanoplanktonic fraction as a source of nutrition for not only aphotoendosymbiotic, but photoendosymbiotic coral colonies. Further to this, experimental studies by Szmant-Froelich and Pilson^52^ conveyed the significance of heterotrophy for *A. poculata*, demonstrating ‘zooxanthellate’ and ‘azooxanthellate’ colonies to have comparable tissue composition given high food availability. However, symbiotic energy contributions have been established as beneficial provided food scarcity^52^, and have since been shown to enact as a supplemental energy source rather than a viable means of sustaining coral tissue biomass alone^43^. Further to this, laboratory studies by Piniak^45^ have indicated similar findings in *O. arbuscula*, identifying heterotrophy to be the main source of energy to the coral host, with photosynthesis enabling increased growth rates. Additional studies by Dimond and Carrington^15^ attributed just 23% of growth rate differences between ‘zooxanthellate’ and ‘azooxanthellate’ colonies to be the result of energy acquired via photosynthesis. However by comparison, additional publications by Leal (2014) and Aichelman et al^24,56^ have shown *O. arbuscula* colonies to rely on photoendosymbiosis in the absence of heterotrophic nutrition, but simultaneously illustrate that heterotrophy may help to mitigate physiological stressors, such as increasing temperatures^56^, once again emphasising the role of heterotrophy for these species irrespective of symbiont state. The limited light availability (which may be biotically influenced through competition with kelp and/or macro-algae^42,43^), and variability of environmental conditions associated with temperate ecosystems, mean temperate corals have an increased reliance on heterotrophically derived nutrition overall^57^.

We further identify significant research gaps surrounding the diversity of shallow water aphotoendosymbiotic and facultatively photoendosymbiotic Scleractinian corals. This is despite shallow-water species comprising approximately 30% of the aphotoendosymbiotic group, and the comparative accessibility of these habitats.

#### Research efforts in the mesophotic zone (30–199 m, encompassing depth variability)

The marine mesophotic zone definably bridges photic and aphotic environments^58^. Recent reviews demonstrate an increased focus on the mesophotic zone within the scientific literature of the last 10 years^58,59^. The mesophotic zone has historically been associated with the facilitation of speciation and evolutionary pathways of marine biota^59^. Additionally, the complexity associated with these systems supports immense biodiversity and unique biological assemblages^58–60^. More recently the mesophotic zone has been considered in terms of providing potential refuge for shallow water species^58,59,61,62^, particularly tropical shallow-water corals in the face of anthropogenic climate change and increasing disturbances^63^. The majority of mesophotic research has been conducted within the tropics, leading to the establishment and knowledge of tropical Mesophotic Coral Ecosystems (MCEs) globally^31,59,62^. An MCE definably consists of light-dependent, and structurally complex, coral communities found bathymetrically between 30 and (sometimes greater than^64,65^) 150 m in depth in tropical to sub-tropical environments^58,62^. Whilst the MCEs are defined by their ability to sustain photosynthesising organisms, specifically photoendosymbiotic Scleractinian corals, they also harbour aphotoendosymbiotic coral communities. These include both hard and soft corals (such as predominate octocoral species) however the latter falls outside the scope of this review. Aphotoendosymbiotic corals are known to become more predominant within the deeper limits of the mesophotic zone^58^. However, despite MCE’s increasing presence within the literature, species identification proves increasingly challenging with depth, resulting in limited knowledge surrounding species biodiversity^58^, and therefore, aphotoendosymbiotic Scleractinian coral diversity within these systems.

The concept of the mesophotic environment has not been well differentiated within temperate and cold regions^59^ with these areas remaining poorly understood despite recent increases in research effort^66,67^. Literature on the bathymetric ranges for mesophotic corals reefs is well resolved within tropical regions (with largely consistent ranges utilised globally)^59^. The upper and lower limits of the mesophotic zone within the tropics have historically been determined by SCUBA restrictions to access the ecosystems for research, and more recently these locations have been defined by light penetration and light influence over photosynthesising organisms’ distribution^59,62^. However, literature surrounding temperate regions is not as consistent, due to several environmental and geographical factors influencing light penetration^59,66^. Geomorphology is also used to define the mesophotic zone, such as the presence of seamounts, sills and slope environments, in addition to biological assemblages and water transparency^59,66^. It has been suggested that the upper and lower limits of the temperate mesophotic zone may be defined by using benthic primary producer abundance^59^, however, the extensive variability of temperate systems results in difficulty establishing set bathymetric ranges for this zone^59^. 25% of total identified publications were found to encompass study sites within the mesophotic depth range as outlined in this review (30–199 m depth). Further, Norway was identified as the location of highest mesophotic research output within the context of this review. This may be attributed to the oceanography of surrounding fjords, which provide habitat to commonly occurring deep water species (such as *Desmophyllum pertusum*)^68^, and increased funding from oil and gas exploration. Of these publications, only 4 titles were identified of relevance to the mesophotic zone specifically within this study^58,60,69,70^. Only one publication was found to specifically address the relevance of the mesophotic habitat specifically^60^. Due to the variability of temperate mesophotic ecosystems, many of the identified publications within this depth category exceed the photic environment despite being situated within the mesophotic zone’s predefined limits. As a result of this, we find extremely limited research on aphotoendosymbiotic or facultatively photoendosymbiotic Scleractinian corals within the mesophotic zone, or more broadly, the understanding of coral distribution and role in mesophotic habitats, highlighting a lack of knowledge surrounding Scleractinian coral assemblages within mesophotic systems globally (supported in findings by Sinniger et al^58^).

#### Research effort for deep-water corals (> 200 m)

Scleractinian corals lacking photoendosymbioses are commonly found within deep, aphotic waters, between 200 and 1000 m depth^10^ and are often described to thrive within temperature ranges of 4–12 degrees^18^. Within high latitude locations these corals are frequently reported between 50 and 1000 m depth, and reports of reef systems at depths of 4000 m have also been recorded at low latitude locations^18^. The distribution of deep-water corals is strongly influenced by oceanography^71^, the presence of hard or rocky substrates, sea mounts and mounds, and in regions with high currents^72^. Reports of deep-water corals date back to the eighteenth century and advances in deep sea technologies have facilitated more intense research effort in the last 2 decades^18^. In the current study deep-water research comprised 50% of the literature identified, although most research is limited to only a few species and locations. Locations identified here with the highest research effort in deep-sea reef systems included United Kingdom/Ireland and Norway (25 publications respectively), where research has focused on *D. pertusum* (included in 85% and 96% of deep-water publications respectively) and *M. oculata* (included in 23% and 15% of publications respectively). This focus is reflected within the global dataset, as *D. pertusum* and *M. oculata* are commonly referred to as foundational species within cold-water or deep-water reef systems^73–75^.

Deep sea reef systems host a diverse array of biological assemblages and are often referred to as biodiversity hotspots comparable to shallow water reef environments^71^. Approximately 20 of the deep sea Scleractinia form structural reef systems^22^, including species such as; *Desmophyllum pertusum*, *Madrepora oculata*, *Oculina varicosa*, *Solenosmilia variabilis, Enallopsammia profunda, Goniocorella dumosa* and *Bathelia candida*, whilst the majority of species records in the deep sea are for solitary species. *Desmophyllum pertusum* (Linneaus 1758) is the most commonly occurring reef-building coral at depth^19^, significantly contributing to reef structures that have been found to span several kilometres^76^. *Desmophyllum pertusum* has a broad geographical range, extending throughout the North Atlantic Ocean^77^ and the Pacific Ocean^78,79^ and can be found anywhere from 50 to 3000 m depth^77^. *D. pertusum* is one of the more intensely studied corals within the deep-sea aphotoendosymbiotic coral literature. We further identify significant research gaps surrounding species diversity and study locations for deep sea habitats.

### Research effort into other symbioses

Bacterial associations are hypothesised to support carbon cycling and nitrogen fixation in deep-water corals^13,80^. Characterisation of the deep-water aphotoendosymbiotic coral microbiome is however relatively recent, and largely limited by the complexities of deep-water sample preservation and analysis. *Desmophyllum pertusum’s* microbiome has however been established within the literature, beginning with Yakimov et al^81^. 8 titles^13,70,81–86^ were found to detail microbial research, 6 of which were found to be relevant to the microbial composition of corals without photoendosymbiosis^13,70,81–83,85^. Bacterial analyses of *D. pertusum* have revealed rich assemblages that differ not only from the surrounding environment, but also between specimens^87^, colourmorphs (red and white varieties)^13^, seasonally^87^ and biogeographical regions^87^. *D. pertusum’s* microbial composition has also been compared to that of *Madrepora oculata*^88^, with *D. pertusum* and *M. oculata* hosting species specific assemblages^87,89^. *M. oculata* exhibited consistent microbiome composition between locations^87,89^ and over time^87^. *Eguchipsammia fistula* has further been found to host niche microbial communities indicative of carbon and nitrogen cycling^82^. As research effort into the diversity of coral associations increases, we further reiterate the importance of maintaining clear and consistent terminology within the published literature.

### Impacts and threats to habitats hosting aphotoendosymbiotic corals

**Figure 8:**
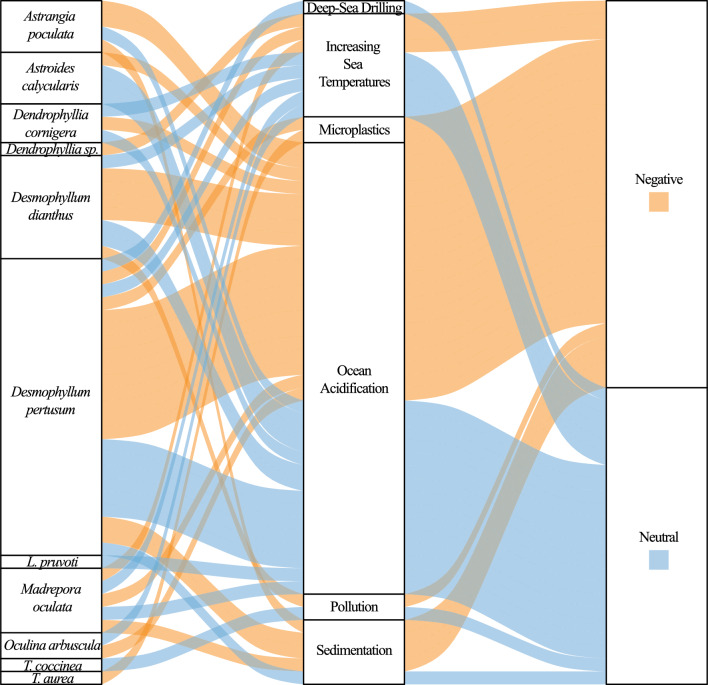
A sankey diagram illustrating the relationship between species, identified threats and stressors, and the reported research outcome. We find a limited number of aphotoendosymbiotic and facultatively photoendosymbiotic species have been considered in terms of anthropogenic threats and stressors.

Oceanic threats associated with anthropogenic climate change, including increasing sea surface temperature and ocean acidification, pollution, and sedimentation, are well documented for photoendosymbiotic Scleractinian coral species^90–92^. However, threats to corals without or with variable photoendosymbiosis remain understudied. Within the context of the search parameters of this review, we find 44 titles of relevance to threats or stressors impacting aphotoendosymbiotic or facultatively symbiotic Scleractinian coral species (Fig. 8) (Supplementary File 1: Threats Data). We find 11 species to be represented within this data, excluding secondary literature.

Of the identified threats (Fig. 8), ocean acidification represented approximately 66% of the identified research effort. Some of these studies suggest aphotoendosymbiotic and facultatively symbiotic coral species to have localised adaptation potential or an increased resilience to lower pH conditions^93,94^. This includes reports that coral calcification rates remain the same between acidified and control experimental treatments^93^ with some coral colonies exhibiting morphological adaptations to changing conditions^94^. Further to this, the plasticity of the aphotoendosymbiotic coral microbiome^95^ and the maintenance of reproductive potential^96^ has also been shown under acidified conditions. However, despite some research presenting the potential resilience of coral species to ocean acidification, other work has illustrated the uncertainty of prolonged exposure to acidified conditions^97^ or species-specific responses^98,99^ highlighting the need for greater research effort and increased species diversity within this space.

The identified publications also investigated; anthropogenic climate change (increasing temperatures)^56,100^, sedimentation^53,101^, pollution^102^ (including microplastics^103,104^) and deep-sea trawling or drilling^105^, as threats to aphotoendosymbiotic and facultatively symbiotic coral species. Despite the potential significance of these threats, there remains a significant lack of species diversity represented within the identified literature.

### Conclusions

Our research demonstrates that significant gaps in our understanding of symbiosis in corals outside of shallow tropical coral reefs remain despite 5 decades of research effort, with research in many ocean habitats limited by logistical complexities of remoteness and high costs, of deep, cold, and remote location research. Under- and unstudied regions are likely areas of high biodiversity and endemism for Scleractinian corals^17^. However, with increasing technological advances, research effort has increased over time and is likely to continue to do so. Given the gradual increase in research, particularly for unexplored locations, our research highlights the importance of standardising terminology and habitat definitions to support ongoing comparative and meta-analysis, and compiling research evidence.

One significant knowledge gap and under-representation of research effort includes the global south. Here we show that research effort across the 482 identified publications has predominately occurred within oceans of the global north (70% of publications). These gaps may be attributed to a comparatively limited access to the necessary assets required for deep-water or mesophotic work. However, almost 20 years ago Cairns’ review of the azooxanthellate Scleractinia of Australia^106^ stated that aphotoendosymbiotic (azooxanthellate) Scleractinian corals likely exhibit high biodiversity and endemism within Australian waters^106^ highlighting the region to be one of the most biodiverse in the world, hosting approximately one third of the known species diversity. Despite the apparent significance of azooxanthellate biodiversity in ocean habitats of Australia (and presumably across the under-explored subtropical, temperate, and polar global south) we find that research effort in Australia has been inconsistent, with a limited understanding of Scleractinian diversity, and its role in ecosystem stability outside of the tropics. Cairns^106^ also highlighted research gaps in the biology, ecology, conservation and management of these species, and researchers have continued to call for a greater research effort, most recently into what is now described as Australia’s Great Southern Reef^107^. Interestingly when compared to the wealth of knowledge surrounding tropical systems, particularly the Great Barrier Reef, research directed towards temperate and cold systems of the Great Southern Reef has been significantly lower^107^. Research publications reporting on corals within Australian exclusive economic zones, including Australian deep-sea locations, predominantly focused on deep- and cold-water reefs, with an increased focus on seamounts, despite the Great Southern Reef (GSR)^107^ extending across the southern coastline of Australia, spanning 5 states, and holding significant ecologic, economic and societal importance^107^. This trend is also mirrored across not only the global south but temperate ecosystems worldwide with the exception of some research hotspots.

Despite comprising approximately half of the known Scleractinian coral diversity, our research demonstrates significant research gaps into aphotoendosymbiotic and facultatively photoendosymbiotic coral species, with much of their associated biology, ecological importance, and threats poorly understood and under-represented within the published literature. Considering the increasing anthropogenic impacts and stressors facing coral populations globally, there is a real risk of losing biodiversity and ecosystem function before we’ve come to understand it.

### Supplementary Information

Supplementary Information 1.

Supplementary Information 2.

Supplementary Information 3.

Supplementary Information 4.

Supplementary Information 5.

## Data Availability

Data and supplementary files are accessible via OSF at: https://osf.io/4fpmx/?view_only=fe7c3f2a8e304e28b9de15e06a3b9a01.
