# Supplementary material for: The global significance of Scleractinian corals without photoendosymbiosis
Source: Sci Rep. 2024 May 3;14:10161. doi: 10.1038/s41598-024-60794-0 (PMC11066124; doi:10.1038/s41598-024-60794-0)
Supplement: Supplementary file 1 — Supplementary Information 1. [file 41598_2024_60794_MOESM1_ESM.pdf]

Beyond Bleached. Systematic Map Methodology and Workflow.

Scoping phase. Landmark publications. Development of search string.

Search String. (("azooxanthellate" OR "deep sea" OR "deep water" OR "temperate" OR "cold water" OR "apozooxan*" OR "facultat* symb*" OR "aposymbio*" OR "aphotic" OR "asymbiotic" OR "non-symbiotic" OR "heterotrophic" OR "non-photosyn*" OR "non-zooxanthella*") AND ("scleractin*") NOT ("soft coral*" OR "octocor*" OR "black coral*" OR "gorgon*" OR "spong*" OR "gastropod*" OR "clam*" OR "anemon*" OR "shrimp*" OR "oyster*" OR "crustacean*" OR "limestone*" OR "fish*" OR "entobia" OR "foramini*" OR "non-scleractinian" OR "bamboo" OR "worm" OR "lobster*" OR "nudibranch*" OR "crinoid*" OR "mollusk*" OR "barnacle*"))

Google Scholar	Web of Science	Scopus	Grey Literature
Top 50	625	556	11

Removal of duplicates, screening for relevance. ("Azooxanthellate, Apozooxanthellate, Facultatively Symbiotic, Asymbiotic, Non-Symbiotic, Non-Zooxanthella(t)e, Non-Photosymbiotic, Deep and Cold").

482 Publications: Final Database.

Data Extraction. Supplemental File 5.
