# Supplementary figures and images for: The global significance of Scleractinian corals without photoendosymbiosis
Source: Sci Rep. 2024 May 3;14:10161. doi: 10.1038/s41598-024-60794-0 (PMC11066124; doi:10.1038/s41598-024-60794-0)

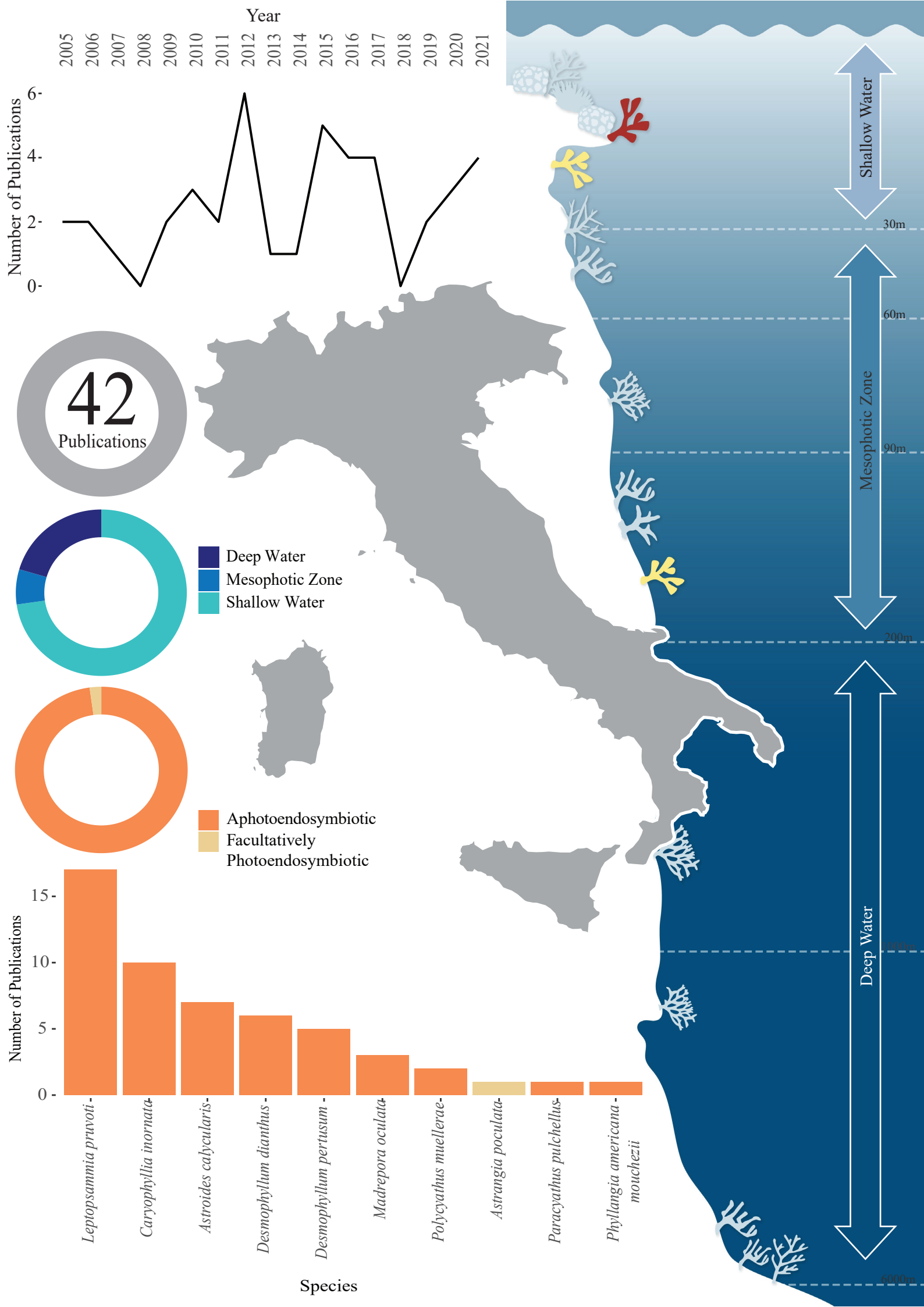

Supplement: Supplementary file 2 — Supplementary Information 2. [file 41598_2024_60794_MOESM2_ESM.pdf]

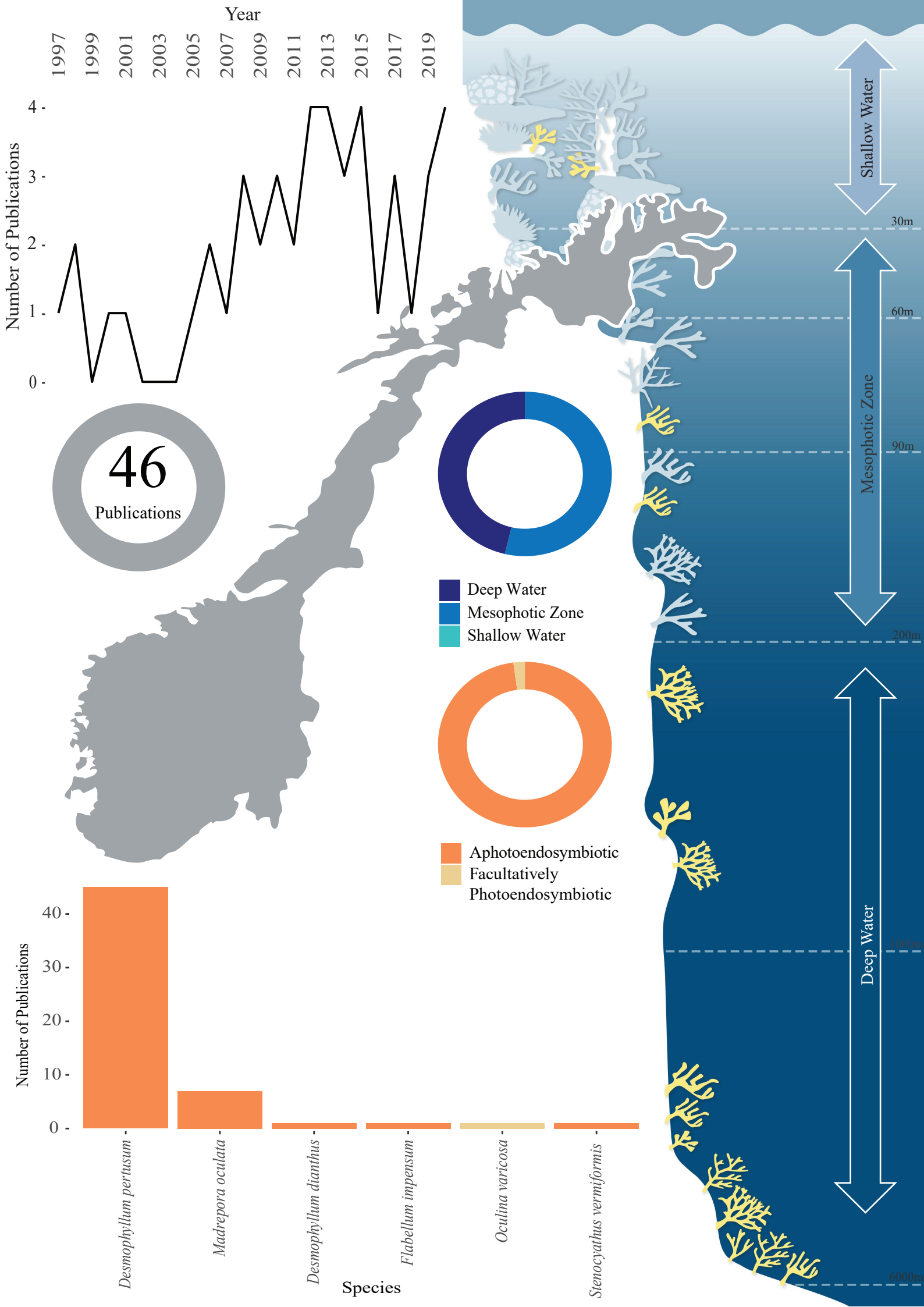

Supplement: Supplementary file 3 — Supplementary Information 3. [file 41598_2024_60794_MOESM3_ESM.pdf]

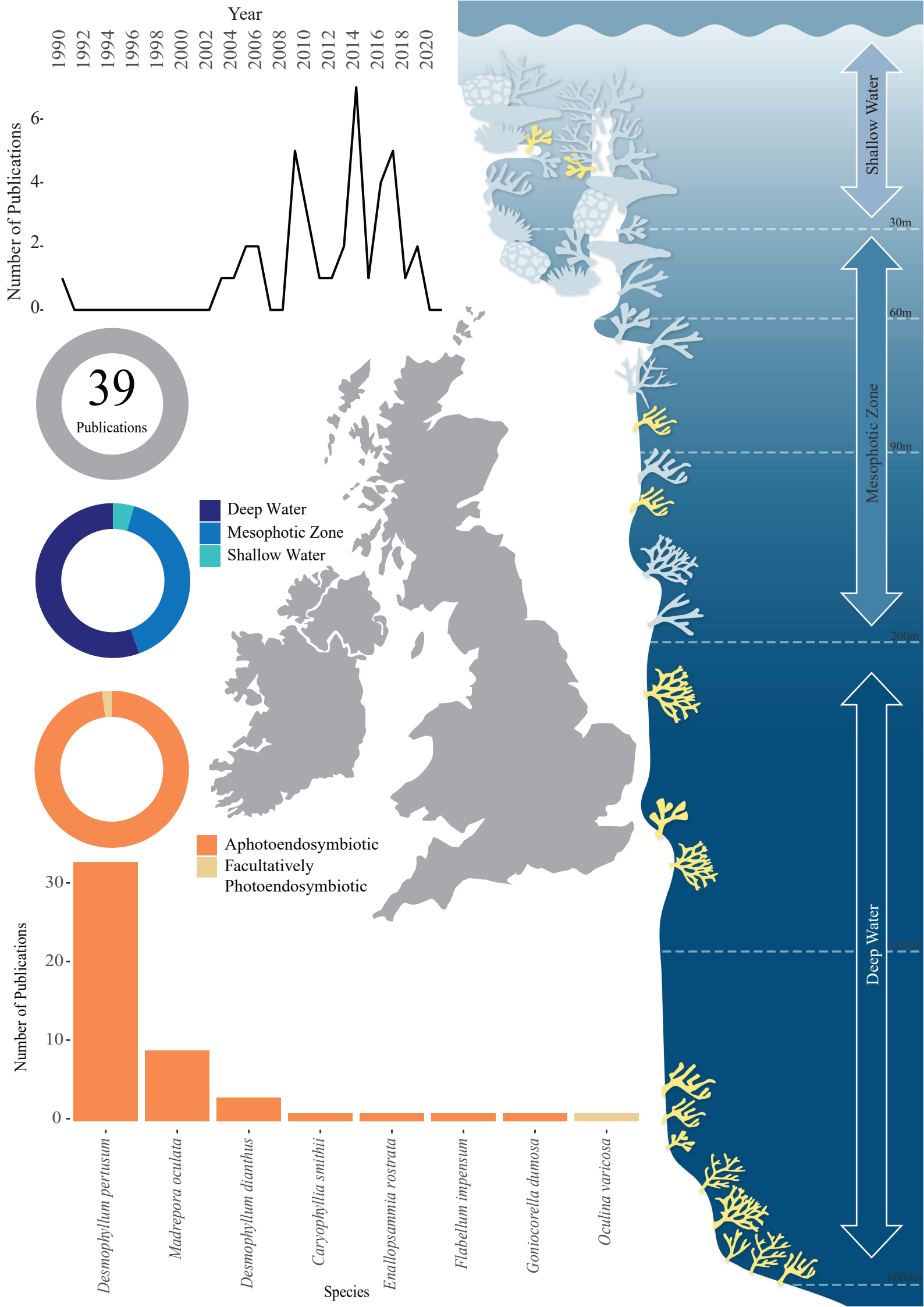

Supplement: Supplementary file 4 — Supplementary Information 4. [file 41598_2024_60794_MOESM4_ESM.pdf]
