# Supplementary material for: The global significance of Scleractinian corals without photoendosymbiosis
Source: Sci Rep. 2024 May 3;14:10161. doi: 10.1038/s41598-024-60794-0 (PMC11066124; doi:10.1038/s41598-024-60794-0)
Supplement: Supplementary file 5 — Supplementary Information 5. [file 41598_2024_60794_MOESM5_ESM.pdf]

Source of Vectors

All case study vectors are sourced from vector download website 'Vecteezy'. Each vector has been downloaded under a Pro license, under which no attribution is required. Please find a list of the vector license numbers below.

Australia Vector Download

License number:

0FC148D2-FC98-4756-BE92-1CE3F791F41F

Italy Vector Download

License number:

2259B819-D1F8-40BE-AE02-336D375E9594

Europe Vector Download

License number:

70882053-EA16-4EC3-941A-3695D28EAAA7

UK and Ireland Vector Download

License number:

69435B9E-1E04-4C2F-B0AB-42482F6D977F

Norway Vector Download

License number:

815E8D3C-9666-4896-99C3-B587D70C506D

USA Vector Download

License number:

32419725-620D-4DCA-8FD4-68FBB7958D54
